# Shifting Public Discourse on Life-Sustaining Treatment in South Korea From 2014 to 2025: Longitudinal Social Media Analysis

**DOI:** 10.2196/95918

**Published:** 2026-09-23

**Authors:** Miryoung Kim, Hae Sun Suh

**Affiliations:** 1 College of Pharmacy, Sunchon National University, 255, Jungang-ro, Suncheon, 57922, Republic of Korea; 2 Research Institute of Life and Pharmaceutical Sciences, Sunchon National University, Suncheon, Republic of Korea; 3 AI Bio Autonomous Research Core Facility Center, Sunchon National University, Suncheon, Republic of Korea; 4 College of Pharmacy, Kyung Hee University, Seoul, Republic of Korea; 5 Department of Regulatory Science, Graduate School, Kyung Hee University, Seoul, Republic of Korea; 6 Institute of Regulatory Innovation through Science, Kyung Hee University, Seoul, Republic of Korea

**Keywords:** life-sustaining treatment, terminal care, palliative care, social media, natural language processing, sentiment analysis, public opinion, health policy

## Abstract

**Background:**

Public discourse plays an important role in shaping how end-of-life care and medical decision-making are understood within society. In South Korea, the implementation of the Life-Sustaining Treatment Decision Act in 2018 established a legal framework for withholding or withdrawing life-sustaining treatment for patients at the end of life. However, relatively little is known about how public discourse surrounding life-sustaining treatment has evolved over time, particularly before and after the act’s implementation.

**Objective:**

This study aimed to examine how online discourse related to life-sustaining treatment in South Korea evolved using large-scale social media data by identifying dominant themes, tracing their temporal changes from 2014 to 2025, and assessing shifts in sentiment before and after the act.

**Methods:**

A total of 16,191 text documents, comprising posts from Naver blogs, Naver cafes, and comments from YouTube, were collected using keywords related to life-sustaining treatment and end-of-life decision-making. Text was preprocessed using the Kiwi tokenizer for Korean morphological analysis, and latent Dirichlet allocation topic modeling was applied to extract underlying themes. A 6-topic solution was selected based on topic coherence and interpretability. Temporal changes in topic prevalence were examined across the study period, and topic distributions were compared between the prelaw (2014‐2017) and postlaw (2018‐2025) periods. Sentiment analysis was conducted using the Kunsan National University Korean sentiment lexicon to classify documents into positive, neutral, and negative categories. A study-defined descriptive net sentiment change index was calculated to summarize changes in positive and negative sentiment following policy implementation.

**Results:**

Six major themes were identified: death with dignity, hospice and palliative care, aging and social care, end-of-life decision-making, euthanasia debate, and withdrawal of treatment. Discourse volume increased more than 17-fold over the study period. Prior to 2018, discussions were dominated by end-of-life decision-making (255/573, 44.5%) and withdrawal of treatment (178/573, 31.1%). Around the time of policy implementation, ethical and legal debates became more prominent. In subsequent years, discourse increasingly reflected care-oriented perspectives, including hospice and palliative care and aging and social care. Sentiment improved most markedly for end-of-life decision-making and hospice and palliative care following the act, whereas aging and social care showed minimal change, and death with dignity exhibited a decline in net sentiment.

**Conclusions:**

Public discourse on life-sustaining treatment in South Korea evolved from a clinical focus to ethical debate and, more recently, to care-oriented perspectives. These changes were temporally associated with the implementation period of the Life-Sustaining Treatment Decision Act but should not be interpreted as causal effects of the legislation. The findings highlight the value of social media data for monitoring policy-associated changes in public discourse on end-of-life care.

## Introduction

End-of-life care and life-sustaining treatment decisions lie at a critical intersection of medicine, ethics, law, and culture. As populations age, questions surrounding the appropriate use of life-prolonging interventions and dignified dying have increasingly entered the public sphere [1,2]. Public discourse on these topics both reflects and shapes societal values, informs policy development, and influences how individuals and families navigate end-of-life decisions [3].

In South Korea, the Life-Sustaining Treatment Decision Act, enacted in 2018, established the first comprehensive national framework for the withholding or withdrawal of life-sustaining treatment at the end of life [4,5]. The act formally recognized patients’ right to autonomous end-of-life decisions; introduced structured mechanisms, including advance directives and a designated decision-making system; and defined the legal conditions under which treatment withdrawal could be authorized, marking a fundamental shift in the institutional approach to end-of-life care in South Korea. Following full implementation in 2018, the actual withdrawal of life-sustaining treatment in clinical settings increased markedly, and public discussion of death, dying, and end-of-life decision-making became more visible in the same period [6-8].

Social media platforms have emerged as important venues for capturing naturalistic public discourse on sensitive health topics [9,10]. Large-scale text data from platforms such as Naver and YouTube, when analyzed using computational methods including topic modeling and sentiment analysis, can reveal shifts in thematic focus and emotional tone that conventional surveys cannot [10-12]. However, despite growing interest in end-of-life attitudes in South Korea, the longitudinal evolution of online discourse—before and after the 2018 act—has received limited scholarly attention [13,14]. Sentiment analysis in health-related social media research can be broadly classified into categorical and dimensional approaches [15]. Categorical sentiment analysis characterizes emotional tone using discrete classes such as positive, neutral, and negative sentiment, whereas dimensional sentiment analysis characterizes affective meaning along continuous dimensions such as valence, arousal, and dominance. Although categorical approaches are useful for interpretable comparisons across large longitudinal corpora, dimensional approaches may provide a more nuanced representation of complex emotional states. This distinction is particularly relevant to end-of-life discourse, where expressions of sadness, fear, relief, acceptance, dignity, and moral concern may coexist in the same discussion.

To address this gap in the longitudinal understanding of online discourse on life-sustaining treatment before and after the act, we analyzed social media data from 2014 to 2025 using topic modeling and lexicon-based sentiment analysis. We aimed to (1) identify dominant themes in public discourse on life-sustaining treatment, (2) trace temporal changes in topic prevalence across the study period, and (3) assess sentiment shifts before and after the implementation of the act. Findings are discussed in a comparative context with end-of-life discourse patterns observed in Western countries.

## Methods

### Study Design

This study used a computational text analysis approach to examine changes in online discourse related to the life-sustaining treatment decision system in South Korea. We focused on how discourse evolved before and after the implementation of the Life-Sustaining Treatment Decision Act in 2018, which introduced a national framework for end-of-life treatment decisions. Drawing on large-scale social media data, we explored changes in both the thematic structure and emotional tone of public discourse over time. The analysis covered the period from January 2014 to December 2025, allowing for a longitudinal comparison of discourse patterns before and after the policy was implemented.

### Data Sources

Text data were collected from 3 widely used Korean online platforms—Naver blogs, Naver cafes, and YouTube comments. Naver is a major Korean web portal and search ecosystem that integrates searches, news, blogs, online communities, maps, shopping, and other services. Because Naver functions not only as a search engine but also as a platform for user-generated content, Naver blogs and Naver cafes have been widely used in South Korea for personal narratives, informational posts, and community-based discussions. Naver blogs generally contain longer personal or informational posts, whereas Naver cafes are topic- or community-based online spaces in which users discuss shared interests, experiences, or concerns. YouTube comments, in contrast, are typically shorter and more reactive, reflecting responses to video content, news, public education materials, or policy-related discussions.

Data were collected using platform-provided APIs rather than direct scraping of private or restricted pages. Naver blog and cafe posts were retrieved using the Naver Open API search end points for blogs and cafe articles, and YouTube videos and comments were retrieved using the YouTube Data API. Searches were conducted using Korean keywords related to life-sustaining treatment and end-of-life decision-making, including terms related to withdrawal or withholding of treatment, hospice and palliative care, death with dignity, euthanasia, and end-of-life decision-making (Table S1 in Multimedia Appendix 1). The platform-specific data collection procedures are summarized in Table S2 in Multimedia Appendix 1.

The representativeness of Naver-based data should be interpreted in light of changes in the Korean online environment during the study period. From 2014 to 2025, online communication in South Korea became increasingly mobile and platform diversified, with greater use of video-based and global social media platforms. Accordingly, Naver blog and cafe data should not be interpreted as representing the Korean general population. Rather, they capture an important segment of Korean-language online discourse, particularly longer-form narrative and community-based discussions on life-sustaining treatment.

For Naver, searches were conducted separately for each platform, keyword, and year. The search query combined each base keyword with the target year, and retrieved records were retained only if the publication date matched the target year. Duplicate Naver records were removed based on URL. For YouTube, keyword-year searches were first used to identify relevant videos through the YouTube Data API. Video metadata, including title, description, publication date, channel title, view count, like count, and comment count, were retrieved when available. Videos were retained only if the upload year matched the target year, and duplicates were removed using video IDs. Top-level comments were then collected from the included videos using the comment thread end point, with a maximum of 30 comments per video ordered by relevance. Videos with disabled comments were skipped, and duplicate comments were removed using comment IDs. Only publicly accessible posts and comments were collected. Usernames and other direct personal identifiers were not analyzed, and results are reported only in aggregate form.

### Data Preprocessing

Prior to analysis, several preprocessing steps were undertaken to prepare the textual data. Duplicate documents and entries not directly related to life-sustaining treatment were removed to ensure the relevance of the dataset. The text was then cleaned by removing URLs, punctuation, special characters, and other nonlinguistic elements.

Given the morphological complexity of the Korean language, we performed morphological analysis using the Kiwi tokenizer to segment sentences into meaningful linguistic units [16]. Tokens corresponding to nouns, verbs, and adjectives were retained, whereas stop words and other function words with limited semantic value were excluded. The resulting tokens were used to construct a document-term matrix, which served as the basis for subsequent topic modeling and sentiment analysis.

### Topic Modeling

To identify dominant themes in online discourse, we applied latent Dirichlet allocation (LDA) topic modeling to the preprocessed corpus [17]. LDA is an unsupervised machine learning approach that identifies latent topics within large text collections by estimating the distribution of words across topics and documents. Candidate LDA models with 1 to 20 topics were evaluated using the coherence metric [18]. Although the 4-topic model showed the highest coherence score (0.4789), the 6-topic model showed a comparable coherence score (0.4754) and provided greater substantive granularity for distinguishing clinically and ethically adjacent themes. Therefore, the 6-topic solution was selected based on both coherence and interpretability. The final model was estimated using the Gensim LdaModel with a random state of 42 and 20 passes. Default alpha and eta settings were used. Sensitivity analyses using alternative random seeds indicated that the major thematic structure of the 6-topic solution was broadly retained. Core themes, including hospice and palliative care, end-of-life decision-making, euthanasia debate, and aging and social care, showed consistent high-probability keywords across runs. Some keyword overlap was observed among conceptually adjacent topics, particularly withdrawal of treatment, end-of-life decision-making, and euthanasia debate, reflecting the close substantive relationship among these themes in end-of-life discourse (Tables S3 and S4 in Multimedia Appendix 1).

Topic labels were assigned through an iterative interpretive review process. The first author reviewed the highest-probability keywords and representative documents for each topic and proposed preliminary labels. The labels were then reviewed and refined through author discussion, with reference to the original Korean text and the substantive meaning of representative documents. The final labels were intended to summarize the dominant thematic meaning of each topic rather than defining mutually exclusive conceptual categories.

### Temporal Analysis

To examine how discourse evolved over time, we analyzed topic prevalence on an annual basis across the study period. Annual topic proportions were calculated by aggregating document-level topic probabilities within each year, allowing us to trace temporal patterns in discourse. Notably, a larger volume of data was collected in more recent years, reflecting the growth of online platform use and increased user activity over time. To account for this imbalance, all temporal analyses were conducted using normalized topic proportions rather than raw document counts.

### Policy Period Comparison

To assess changes in discourse associated with the implementation of the Life-Sustaining Treatment Decision Act, we divided the dataset into 2 periods: before implementation (2014‐2017) and after implementation (2018‐2025). Topic distributions were compared between these periods to examine shifts in the thematic focus of online discussions following the introduction of the life-sustaining treatment decision system.

### Sentiment Analysis and Change Assessment

We adopted a categorical lexicon-based sentiment approach, classifying the overall emotional tone of each document into positive, neutral, or negative categories. This approach was selected because it provides a transparent and reproducible framework for comparing aggregate sentiment patterns across topics and periods in a large Korean-language social media corpus. A range of lexicon-based sentiment resources has been developed for different languages and affective representations, including English-language polarity lexicons such as the Semantic Orientation Calculator and AFINN; dimensional affective lexicons such as the Affective Norms for English Words and its extended version, the National Research Council Canada Valence, Arousal, and Dominance Lexicon; and language-specific resources such as the Chinese EmoBank [19-24]. However, these resources are not directly applicable to Korean-language social media text because sentiment-bearing expressions are language and culture dependent. Applying non-Korean lexicons would require translation or cross-lingual mapping, which may alter affective meaning, particularly in culturally sensitive discourse on death, dignity, family caregiving, and end-of-life decision-making. Therefore, we used the KNU Korean sentiment lexicon, a Korean-language sentiment resource, to classify sentiment in a manner consistent with the linguistic characteristics of the corpus [25].

The KNU Korean sentiment lexicon is a general-purpose Korean sentiment resource and has not been specifically developed or validated for end-of-life care discourse. Accordingly, sentiment classification in this study was interpreted as a descriptive indicator of aggregate emotional tone rather than as a direct measure of individual affective states.

To summarize topic-specific sentiment changes between the pre- and postimplementation periods, we calculated a study-defined descriptive net sentiment change index. For each topic, the index was defined as (positivepost – positivepre) – (negativepost – negativepre), where “positivepost” and “positivepre” indicate the percentage of documents classified as positive in the post- and preimplementation periods, respectively, and “negativepost” and “negativepre” indicate the corresponding percentages of documents classified as negative in the post- and preimplementation periods, respectively. Positive values, therefore, indicate an increase in positive sentiment, a decrease in negative sentiment, or both. Negative values indicate a decrease in positive sentiment, an increase in negative sentiment, or both. This index was used as a descriptive summary of direction and magnitude rather than as an externally validated psychometric measure.

All analyses, including data preprocessing, topic modeling, temporal analysis, and sentiment analysis, were conducted using Python (version 3.14.3; Python Software Foundation).

### Ethical Considerations

This study used publicly available, deidentified data and did not involve human participants as defined by institutional guidelines. Therefore, institutional review board approval was not required.

## Results

### Overview of the Dataset

A total of 16,191 social media documents related to life-sustaining treatment were collected from Naver and YouTube between 2014 and 2025 after preprocessing. The volume of discourse increased markedly over time. The number of posts rose from 178 in 2014 to 3160 in 2025, representing a more than 17-fold increase. A substantial rise was observed after 2020, with annual counts exceeding 500 posts from 2021 onward and peaking in 2025. The dataset was heavily concentrated in the postpolicy period. Of the 16,191 total documents, 15,618 (96.5%) were posted after the implementation of the Life-Sustaining Treatment Decision Act (2018‐2025), whereas only 573 (3.5%) were from the prepolicy period (2014‐2017). In terms of platform distribution, YouTube comments accounted for 52.6% (n=8513) of the documents, whereas Naver blogs and cafes constituted 47.4% (n=7678) of the documents. Notably, prepolicy data were drawn exclusively from Naver blogs, whereas postpolicy data included both platforms, reflecting the increasing role of video-based platforms in public discourse over time.

### Topic Characteristics

**Figure 1.**
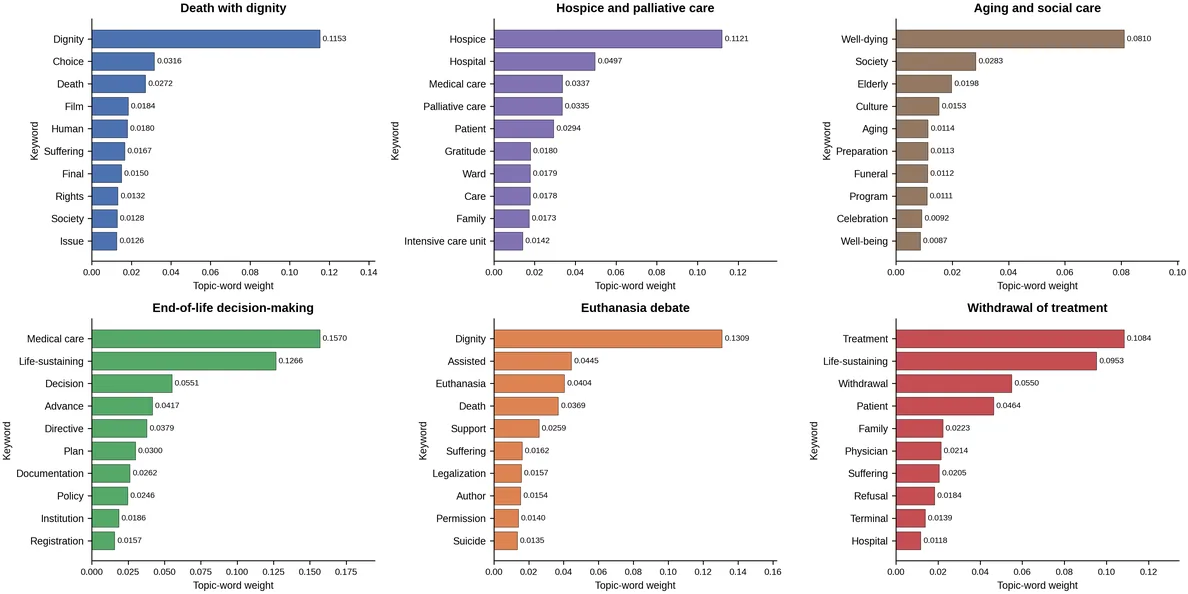
Top 10 keywords and their weights for each of the 6 identified topics in online discourse on life-sustaining treatment in South Korea (2014‐2025).

LDA identified 6 distinct topics representing major dimensions of public discourse: death with dignity, hospice and palliative care, aging and social care, end-of-life decision-making, euthanasia debate, and withdrawal of treatment (Figure 1). Each topic was characterized by a coherent set of frequently co-occurring keywords. Some keywords were shared across topics, reflecting their use in multiple contexts within end-of-life discourse.

The death with dignity topic was primarily associated with high-weight terms such as “dignity,” “choice,” and “death,” reflecting discussions centered on autonomy and the right to self-determined end-of-life decisions. The hospice and palliative care topic was dominated by terms such as “hospice,” “hospital,” and “palliative care,” indicating a focus on care provision in clinical settings. The aging and social care topic included high-weight terms such as “well-dying,” “society,” and “elderly,” suggesting a broader societal perspective on aging and end-of-life preparation. The end-of-life decision-making topic was characterized by terms such as “medical care,” “life-sustaining,” and “decision,” reflecting institutional and procedural aspects of treatment decision-making. The euthanasia debate topic was defined by terms such as “dignity,” “assisted,” and “euthanasia,” indicating ongoing ethical and legal debates surrounding assisted death. Finally, the withdrawal of treatment topic was associated with terms such as “treatment,” “life-sustaining,” and “withdrawal,” highlighting discussions related to the discontinuation of medical interventions. As the analysis was conducted using Korean-language data, the extracted keywords were translated into English to facilitate interpretation and presentation. The full list of Korean-English keyword mappings for each topic is provided in Table S2 in Multimedia Appendix 1.

### Temporal Trends in Topic Prevalence

**Figure 2.**
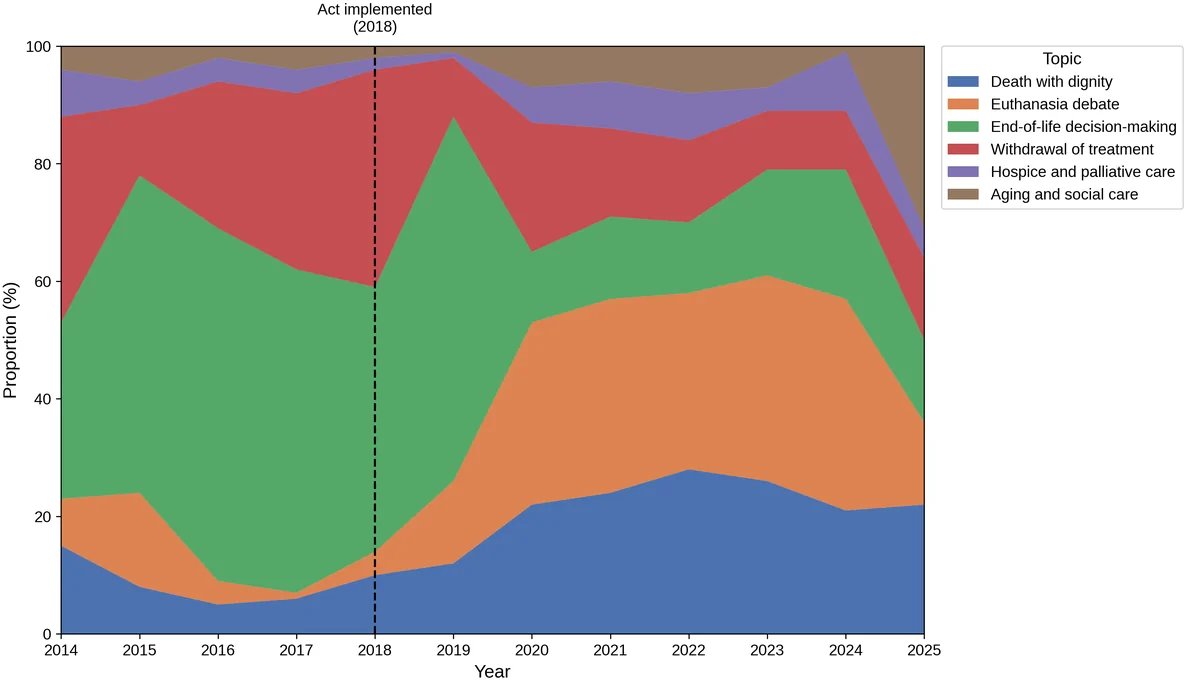
Temporal changes in topic proportions. The dashed line indicates the implementation of the Life-Sustaining Treatment Decision Act in 2018.

Temporal analysis revealed substantial shifts in topic prevalence over the study period (Figure 2). Prior to 2018, discourse was dominated by the end-of-life decision-making and withdrawal of treatment topics, which together accounted for most discussions, often exceeding 60% to 70% of total topic proportions. Around 2018, a marked transition was observed. The relative dominance of the end-of-life decision-making topic declined, whereas discussions on topics related to death with dignity and euthanasia debate increased, indicating a shift toward ethical and normative discussions during the policy implementation period. From 2020 onward, the discourse became more diversified. The proportion of hospice and palliative care and aging and social care topics increased steadily, whereas the previously dominant clinical topics declined. By the later years, discourse was more evenly distributed across topics, with care-oriented and social themes occupying a larger share. These patterns indicate a transition from clinically focused discussions toward broader ethical debates and, subsequently, toward care-oriented and social perspectives.

### Changes in Topic Distribution Before and After Policy Implementation

**Figure 3.**
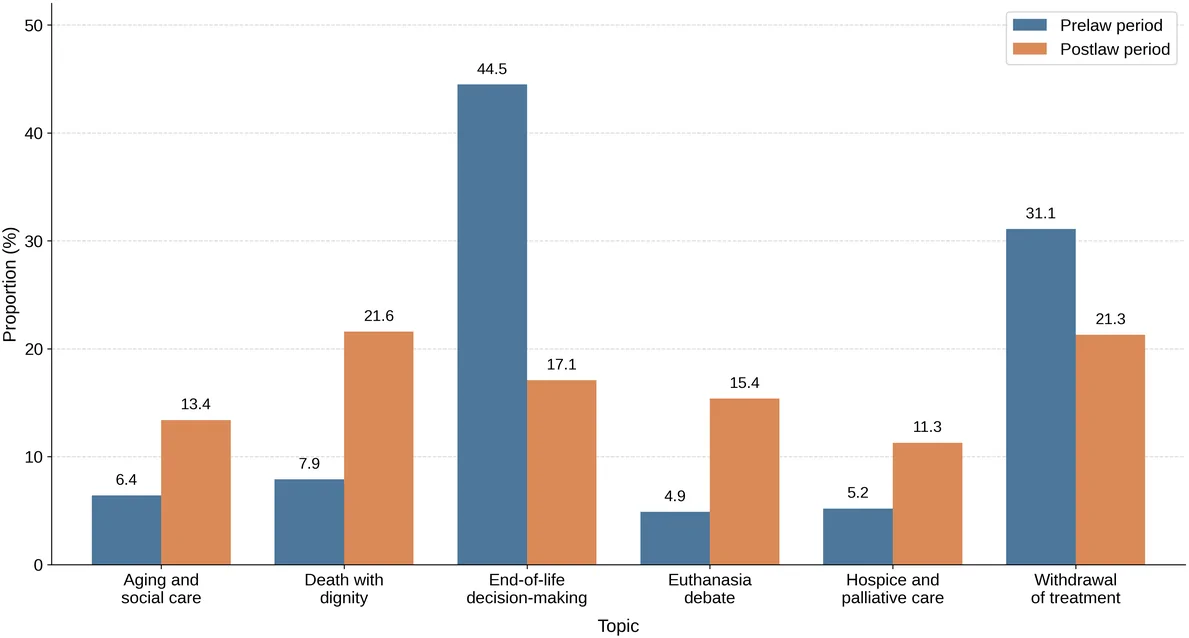
Topic distribution before and after implementation of the Life-Sustaining Treatment Decision Act. Proportions are compared between the prelaw (2014‐2017) and postlaw (2018‐2025) periods.

Comparisons between the prelaw (2014‐2017) and postlaw (2018‐2025) periods further illustrate these shifts (Figure 3). In the prelaw period, end-of-life decision-making was the most prominent topic, accounting for the largest share of discourse, followed by withdrawal of treatment. In contrast, the postlaw period showed a clear redistribution of topic prevalence. The share of end-of-life decision-making decreased substantially (255/573, 44.5% to 2671/15,618, 17.1%), whereas death with dignity showed the largest relative increase (45/573, 7.9% to 3373/15,618, 21.6%), followed by euthanasia debate (28/573, 4.9% to 2405/15,618, 15.4%) and hospice and palliative care (30/573, 5.2% to 1765/15,618, 11.3%). These changes indicate temporal shifts in online discourse around and after the implementation period of the act from a narrower focus on clinical decision-making toward broader discussions encompassing ethical considerations, care practices, and social contexts.

### Sentiment Distribution and Changes After Policy Implementation

**Figure 4.**
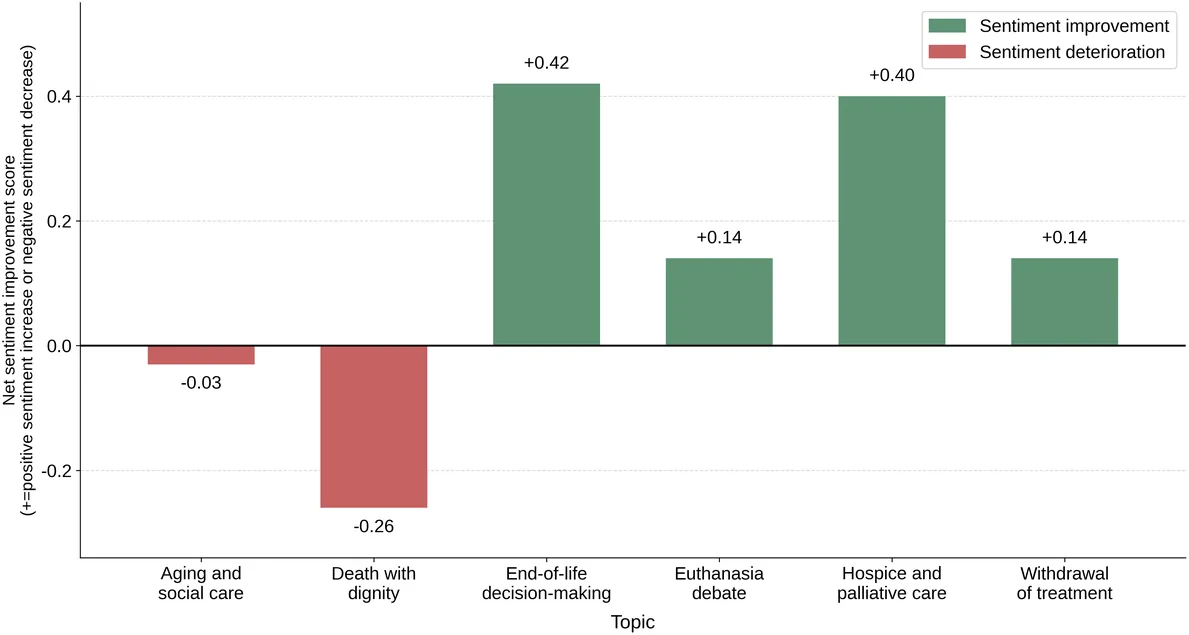
Descriptive net sentiment change index by topic. Positive values indicate an increase in positive sentiment or a decrease in negative sentiment following the act.

To examine sentiment changes following the implementation of the Life-Sustaining Treatment Decision Act, the descriptive net sentiment change index was calculated for each topic (Figure 4). The magnitude and direction of sentiment change varied across topics. The largest positive shifts were observed in end-of-life decision-making and hospice and palliative care, both of which showed increases in positive sentiment combined with reductions in negative sentiment. More moderate positive shifts were observed in euthanasia debate and withdrawal of treatment, whereas aging and social care showed minimal change in sentiment, indicating limited shifts in the overall emotional tone of these discussions. In contrast, death with dignity was the only topic that exhibited a negative shift in the index, which appears to be primarily attributable to an increase in negative sentiment, as illustrated in Figure S1 in Multimedia Appendix 1.

## Discussion

### Principal Findings

This longitudinal social media analysis found substantial changes in the volume, thematic composition, and aggregate sentiment of online discourse on life-sustaining treatment in South Korea between 2014 and 2025. These changes were most apparent around and after the implementation period of the Life-Sustaining Treatment Decision Act, but they should be interpreted as temporal associations rather than causal effects of the legislation. Prior to 2018, discussions were largely centered on clinical aspects of care, particularly end-of-life decision-making and withdrawal of treatment. Around the time of implementation of the Life-Sustaining Treatment Decision Act, ethical topics such as death with dignity and euthanasia debate became more prominent. In subsequent years, discourse increasingly reflected care-oriented and broader social perspectives, including hospice and palliative care and aging and social care. These findings suggest that the implementation of the Life-Sustaining Treatment Decision Act was associated not only with changes in the scope of medical decision-making but also with broader shifts in the structure of public discourse.

Previous studies reporting an increase in the actual implementation of life-sustaining treatment withdrawal in clinical practice following the act provide empirical context for the observed increase in discourse volume in this study [6]. In addition, this study found that sentiment toward end-of-life decision-making and hospice and palliative care became more positive over time. This pattern may reflect increasing social acceptance as these domains became more clearly defined within a legal and institutional framework. Park et al [8] suggested that the concept of well-dying in South Korea extends beyond individual preparation for death and is closely linked to family burden and social care. Consistent with this, the increasing prominence of “family” as a high-weight keyword in both the withdrawal of treatment and hospice and palliative care topics indicates that life-sustaining treatment is increasingly framed not only as a medical decision but also as a broader social care issue. However, the minimal change in sentiment observed for aging and social care suggests that these issues continue to be perceived as structural burdens and that policy implementation alone may be insufficient to substantially shift public perceptions in this domain.

The results also showed an increase in discourse related to death with dignity and euthanasia debate following policy implementation, suggesting that the introduction of the legal framework contributed to expanding these topics into the public sphere. The observed reduction in negative sentiment toward euthanasia debate is consistent with prior survey-based findings indicating relatively high public support for euthanasia legalization in South Korea [14]. In contrast, death with dignity showed an increase in negative sentiment. This may be explained by the conceptual ambiguity of the term “death with dignity,” which is not a strictly defined legal concept and is often used in multiple ways in public discourse. As a result, it may be interpreted differently from the legally defined concept of life-sustaining treatment withdrawal, leading to increased debate and heightened emotional tension.

The trajectory of discourse on life-sustaining treatment in South Korea appears to differ from that observed in many Western countries. In Western contexts, discussions on end-of-life care have developed gradually over time, driven by bioethics, patient rights, and the expansion of palliative care, and may be described as a discourse-driven model [3,26,27]. In contrast, the Korean case may be better understood as a policy-associated discourse shift in which legislative implementation and institutional change coincided with increased public engagement with a previously marginalized topic [28]. This framing emphasizes the temporal and contextual relationship between policy implementation and online discourse while avoiding causal interpretation.

Differences are also evident in the structure of decision-making reflected in the discourse. Whereas end-of-life decision-making in Western contexts is often centered on individual autonomy, the discourse observed in this study reflects a stronger emphasis on family involvement [29]. Previous research has shown that a substantial proportion of life-sustaining treatment decisions involve family members in South Korea, and in this study, family-related keywords were frequently identified in both the withdrawal of treatment and hospice and palliative care topics [6]. This pattern reflects the influence of family-centered cultural norms in Asian contexts, where major life decisions, including those at the end of life, are often made collectively [30].

This study has several strengths. First, it analyzed a large-scale dataset of social media content spanning more than a decade (2014‐2025), allowing for a comprehensive examination of longitudinal changes in public discourse on life-sustaining treatment. Second, by integrating topic modeling, temporal analysis, and sentiment analysis, this study was able to capture both the thematic structure and emotional orientation of discourse, providing a multidimensional understanding of how public discussions evolved over time. Third, the use of multiple platforms, including Naver blogs, Naver cafes, and YouTube comments, enabled the analysis to reflect diverse forms of user-generated content, ranging from personal narratives and community discussions to public reactions to policy issues. Fourth, this study offers a conceptual contribution by characterizing the Korean experience as a policy-triggered discourse model, in contrast to the discourse-driven trajectories observed in Western contexts. This distinction provides a framework that may be applicable to other Asian societies that have undergone similar legislative reforms in end-of-life care—including Taiwan’s Patient Right to Autonomy Act and Japan’s ongoing policy deliberations—and invites comparative inquiry into whether legislative enactment similarly catalyzes public discourse in these contexts [31,32].

However, several limitations should be considered when interpreting the findings. First, the data were derived from selected online platforms and may not be representative of the general population as social media users differ in demographic and behavioral characteristics. Platform-specific differences should also be considered. Blogs, cafes, and YouTube comments differ in text length, user motivation, community structure, and degree of reactivity to media content. Therefore, the combined corpus should not be interpreted as a representative measure of public opinion in the general Korean population. Rather, it reflects discourse patterns within selected online environments where end-of-life issues were publicly discussed. Second, the volume of data was substantially higher in the postimplementation period than in the preimplementation period, which may have influenced the stability of topic estimation and sentiment comparisons despite the use of normalized proportions in temporal analyses. Third, although sensitivity analyses suggested that the major thematic structure of the 6-topic LDA solution was broadly retained across alternative random seeds, some keyword overlap was observed among conceptually adjacent themes. Topic labels should therefore be interpreted as dominant thematic summaries rather than mutually exclusive conceptual categories. Fourth, sentiment analysis was conducted using a general-purpose lexicon-based approach, which may not fully capture the contextual nuances of Korean end-of-life discourse. End-of-life discussions often contain emotionally ambiguous and context-dependent expressions. For example, narratives about withdrawal of treatment, death with dignity, or hospice care may include negative lexical items while conveying relief, acceptance, respect for patient autonomy, or support for dignified dying. Conversely, apparently neutral statements may reflect moral concern, anxiety, or distrust. Therefore, the sentiment estimates in this study should be interpreted as aggregate lexicon-based indicators of emotional tone rather than definitive classifications of subjective emotion. Future research should validate sentiment classification using manually annotated Korean end-of-life discourse and develop domain-adapted or context-aware sentiment models, including dimensional or aspect-based approaches such as multidimensional relation models and recent shared-task initiatives in dimensional aspect-based sentiment analysis [33,34]. Fifth, this study was observational and descriptive in nature; therefore, the findings cannot establish a causal effect of the Life-Sustaining Treatment Decision Act on public discourse. Although thematic and sentiment changes were observed around the implementation period of the act, other social and historical factors may also have contributed to these trends. These include population aging, increasing public awareness of hospice and palliative care, media coverage of end-of-life issues, changes in platform use, COVID-19–related experiences with serious illness and death, and broader public discussion of advance directives. Accordingly, the results should be interpreted as policy-associated temporal shifts in online discourse rather than evidence of a direct policy effect.

### Conclusions

Public discourse on life-sustaining treatment in South Korea underwent substantial structural changes following the implementation of the Life-Sustaining Treatment Decision Act in 2018, shifting from a clinical focus to ethical debates and, subsequently, to care-oriented and social perspectives. Sentiment toward end-of-life decision-making and hospice and palliative care became more positive over time, whereas aging and social care showed minimal change in sentiment, indicating that structural challenges in this domain remain insufficiently addressed by policy alone. These findings should be interpreted as policy-associated temporal changes rather than causal effects of the act. They highlight the importance of integrating social and familial dimensions into public health communication and advance care planning and underscore the value of social media data in monitoring policy-associated changes in public discourse.

## Supplementary material

10.2196/95918 Multimedia Appendix 1 Supplementary materials comprising data collection keywords, platform-specific data collection procedure, topic number selection using c_v coherence scores, latent Dirichlet allocation random-seed sensitivity analysis, Korean–English translation of topic keywords, and change in positive and negative sentiment proportions after the act, by topic.

## Abbreviations

LDA: latent Dirichlet allocation
