# Supplementary material for: Shifting Public Discourse on Life-Sustaining Treatment in South Korea From 2014 to 2025: Longitudinal Social Media Analysis
Source: JMIR Infodemiology. 2026 Sep 23;6:e95918. doi: 10.2196/95918 (PMC13600655; doi:10.2196/95918)
Supplement: Supplementary file 1 [file infodemiology-v6-e95918-s001.docx]

**Supplementary Table S1.** Keywords Used for Social Media Data Collection

| **No.** | **Korean Keyword** | **English Translation** | **Thematic Category** |
| --- | --- | --- | --- |
| 1 | 연명치료 중단 | Withdrawal of life-sustaining treatment | Life-sustaining treatment |
| 2 | 연명의료결정 | Life-sustaining treatment decision | Life-sustaining treatment |
| 3 | 사전연명의료의향서 | Advance directive for life-sustaining treatment | Life-sustaining treatment |
| 4 | 연명의료계획서 | Life-sustaining treatment care plan | Life-sustaining treatment |
| 5 | 존엄사 | Death with dignity / Euthanasia | End-of-life ethics |
| 6 | DNR | Do-Not-Resuscitate | Clinical decision-making |
| 7 | 호스피스 | Hospice (care) | Palliative and hospice care |
| 8 | 웰다잉 | Well-dying | Aging and social care |

All keywords were used for data collection from Naver Blog, Naver Cafe, and YouTube covering the period January 2014 to December 2025. Keywords were selected to capture commonly used Korean-language expressions related to life-sustaining treatment and end-of-life decision-making.

**Supplementary Table S2. Platform-specific data collection procedure**

| **Platform** | **Source/API** | **Search unit** | **Date filtering** | **Duplicate removal** | **Text field used** |
| --- | --- | --- | --- | --- | --- |
| Naver Blog | Naver Open API blog search endpoint | Keyword-year query | Publication year matched to target year | URL | Title and description/post text available through API |
| Naver Cafe | Naver Open API cafe article search endpoint | Keyword-year query | Publication year matched to target year | URL | Title and description/article text available through API |
| YouTube comments | YouTube Data API search, videos, and commentThreads endpoints | Keyword-year video search followed by top-level comment retrieval | Video upload year matched to target year | Video ID and comment ID | Top-level comments from included videos |

**Supplementary Figure S1**

**Supplementary Table S3. Topic number selection using c_v coherence scores**

| **Number of topics** | **c_v coherence score** |
| --- | --- |
| 1 | 0.3510 |
| 2 | 0.4655 |
| 3 | 0.4492 |
| 4 | 0.4789 |
| 5 | 0.4336 |
| 6 | 0.4754 |
| 7 | 0.4551 |
| 8 | 0.4730 |
| 9 | 0.4399 |
| 10 | 0.4340 |
| 11 | 0.4305 |
| 12 | 0.4284 |
| 13 | 0.4365 |
| 14 | 0.4324 |
| 15 | 0.4438 |
| 16 | 0.4162 |
| 17 | 0.4002 |
| 18 | 0.4317 |
| 19 | 0.4171 |
| 20 | 0.4277 |

Note. Although the four-topic model had the highest c_v coherence score, the six-topic model had a comparable score and provided greater substantive granularity for distinguishing clinically and ethically adjacent themes.

**Supplementary Table S4. LDA random-seed sensitivity analysis**

| **Alternative seed** | **Mean top-keyword Jaccard vs seed 42** | **Median top-keyword Jaccard vs seed 42** | **Minimum topic Jaccard vs seed 42** | **Mean overlap among top 15 keywords** | **Dominant-topic agreement vs seed 42** |
| --- | --- | --- | --- | --- | --- |
| 1 | 0.389 | 0.432 | 0.000 | 7.83 | 47.8% |
| 100 | 0.461 | 0.579 | 0.000 | 8.83 | 51.1% |
| 2025 | 0.395 | 0.429 | 0.111 | 8.00 | 51.7% |

Note. The reference model used random seed 42. Sensitivity analyses used the same preprocessing pipeline, dictionary, and six-topic specification. Results indicate that the major thematic structure was broadly retained, although some overlap was observed among conceptually adjacent topics.

**Supplementary Table S5.** Korean–English Translation of Topic Keywords Identified by LDA Topic Modeling

Korean–English translation of keywords identified in the topic modeling analysis. Korean tokens extracted from social media texts were translated into English to facilitate interpretation and presentation of the topic keywords.

| **No.** | **Topic** | **Korean keyword** | **English translation** |
| --- | --- | --- | --- |
| 1 | Death with dignity | 존엄 | Dignity |
| 2 | Death with dignity | 선택 | Choice |
| 3 | Death with dignity | 죽음 | Death |
| 4 | Death with dignity | 영화 | Film |
| 5 | Death with dignity | 인간 | Human |
| 6 | Death with dignity | 고통 | Suffering |
| 7 | Death with dignity | 마지막 | Final |
| 8 | Death with dignity | 권리 | Rights |
| 9 | Death with dignity | 우리 | Society |
| 10 | Death with dignity | 문제 | Issue |
| 11 | Hospice and palliative care | 호스피스 | Hospice |
| 12 | Hospice and palliative care | 병원 | Hospital |
| 13 | Hospice and palliative care | 의료 | Medical care |
| 14 | Hospice and palliative care | 완화 | Palliative care |
| 15 | Hospice and palliative care | 환자 | Patient |
| 16 | Hospice and palliative care | 감사 | Gratitude |
| 17 | Hospice and palliative care | 병동 | Ward |
| 18 | Hospice and palliative care | 요양 | Care |
| 19 | Hospice and palliative care | 가족 | Family |
| 20 | Hospice and palliative care | 중환자실 | Intensive care unit |
| 21 | Aging and social care | 웰다잉 | Well-dying |
| 22 | Aging and social care | 사회 | Society |
| 23 | Aging and social care | 노인 | Elderly |
| 24 | Aging and social care | 문화 | Culture |
| 25 | Aging and social care | 고령 | Aging |
| 26 | Aging and social care | 준비 | Preparation |
| 27 | Aging and social care | 장례 | Funeral |
| 28 | Aging and social care | 프로그램 | Program |
| 29 | Aging and social care | 축하 | Celebration |
| 30 | Aging and social care | 웰빙 | Well-being |
| 31 | End-of-life decision-making | 의료 | Medical care |
| 32 | End-of-life decision-making | 연명 | Life-sustaining |
| 33 | End-of-life decision-making | 결정 | Decision |
| 34 | End-of-life decision-making | 사전 | Advance |
| 35 | End-of-life decision-making | 의향서 | Directive |
| 36 | End-of-life decision-making | 계획서 | Plan |
| 37 | End-of-life decision-making | 작성 | Documentation |
| 38 | End-of-life decision-making | 제도 | Policy |
| 39 | End-of-life decision-making | 기관 | Institution |
| 40 | End-of-life decision-making | 등록 | Registration |
| 41 | Euthanasia debate | 존엄 | Dignity |
| 42 | Euthanasia debate | 조력 | Assisted |
| 43 | Euthanasia debate | 안락사 | Euthanasia |
| 44 | Euthanasia debate | 죽음 | Death |
| 45 | Euthanasia debate | 찬성 | Support |
| 46 | Euthanasia debate | 고통 | Suffering |
| 47 | Euthanasia debate | 합법 | Legalization |
| 48 | Euthanasia debate | 저자 | Author |
| 49 | Euthanasia debate | 허용 | Permission |
| 50 | Euthanasia debate | 자살 | Suicide |
| 51 | Withdrawal of treatment | 치료 | Treatment |
| 52 | Withdrawal of treatment | 연명 | Life-sustaining |
| 53 | Withdrawal of treatment | 중단 | Withdrawal |
| 54 | Withdrawal of treatment | 환자 | Patient |
| 55 | Withdrawal of treatment | 가족 | Family |
| 56 | Withdrawal of treatment | 의사 | Physician |
| 57 | Withdrawal of treatment | 고통 | Suffering |
| 58 | Withdrawal of treatment | 거부 | Refusal |
| 59 | Withdrawal of treatment | 말기 | Terminal |
| 60 | Withdrawal of treatment | 병원 | Hospital |
